# Bone Density and Texture from Minimally Post-Processed Knee Radiographs in Subjects with Knee Osteoarthritis

**DOI:** 10.1007/s10439-019-02227-y

**Published:** 2019-02-14

**Authors:** Jukka Hirvasniemi, Jaakko Niinimäki, Jérôme Thevenot, Simo Saarakkala

**Affiliations:** 1 Center for Machine Vision and Signal Analysis, Faculty of Information Technology and Electrical Engineering, University of Oulu, PO Box 4500, 90014 Oulu, Finland; 2 Department of Diagnostic Radiology, Oulu University Hospital, Oulu, Finland; 3 Research Unit of Medical Imaging, Physics and Technology, Faculty of Medicine, University of Oulu, Oulu, Finland

**Keywords:** Radiography, Osteoarthritis, Knee, Bone texture, Bone density, Bone marrow lesion

## Abstract

Plain radiography is the most common modality to assess the stage of osteoarthritis. Our aims were to assess the relationship of radiography-based bone density and texture between radiographs with minimal and clinical post-processing, and to compare the differences in bone characteristics between controls and subjects with knee osteoarthritis or medial tibial bone marrow lesions (BMLs). Tibial bone density and texture was evaluated from radiographs with both minimal and clinical post-processing in 109 subjects with and without osteoarthritis. Bone texture was evaluated using fractal signature analysis. Significant correlations (*p *< 0.001) were found in all regions (between 0.94 and 0.97) for calibrated bone density between radiographs with minimal and clinical post-processing. Correlations varied between 0.51 and 0.97 (*p *< 0.001) for FD_Ver_ texture parameter and between − 0.10 and 0.97 for FD_Hor_. Bone density and texture were different (*p *< 0.05) between controls and subjects with osteoarthritis or BMLs mainly in medial tibial regions. When classifying healthy and osteoarthritic subjects using a machine learning-based elastic net model with bone characteristics, area under the receiver operating characteristics (ROCAUC) curve was 0.77. For classifying controls and subjects with BMLs, ROCAUC was 0.85. In conclusion, differences in bone density and texture can be assessed from knee radiographs when using minimal post-processing.

**Electronic supplementary material:**

The online version of this article (10.1007/s10439-019-02227-y) contains supplementary material, which is available to authorized users.

## Introduction

Osteoarthritis (OA) is the most common degenerative joint disease and it causes a large economic burden to the society as the direct and indirect costs can reach as high as 2.5% of the gross domestic product of a nation,^21^ not to mention the reduction of the quality of life of an individual. OA-related changes in the subchondral bone include bone sclerosis (hardening of bone), osteophytes, bone cysts, and bone deformation.^4^

Plain radiography is a cheap, fast, and widely available imaging method. It is especially suitable for imaging of bone tissue. Plain radiographs are commonly used in diagnostics of diseases that affect bone density and structure, such as OA. Due to the aforementioned advantages of the plain radiography, development of image analysis tools for the assessment of OA-related changes is of interest. However, efforts are needed to produce comparable plain radiographs between X-ray imaging systems from different manufacturers, as image acquisition settings and post-processing (PP) algorithms affect the appearance of the final image and the assessment of bone density.^14^ Typical clinical PP algorithms apply non-linear filtering and adjustment on contrast curves of an image to improve diagnostic readability.^14^ In many cases, a regular user does not have an access on details of the PP method and parameters. To overcome the issue with quantitative image analyses, calibration of the grayscale values in an image using an aluminum step wedge has been proposed.^9^,^14^,^22^,^34^

We have recently shown that bone texture assessed from radiographs differs between subjects with and without bone marrow lesions (BMLs).^7^ However, that study did not assess bone density due to the lack of a calibration object in images and the bone texture was calculated only from two regions of interests (ROIs) in medial tibia. Recently, multiple ROIs covering the majority of the proximal tibia area have been proposed to address this limitation.^11^,^12^

In theory, texture analysis of bone is not as dependent on the imaging conditions as the direct evaluation of grayscale values. However, because clinical PP algorithm enhances edges in an image, it may still affect texture of the processed image. In OA research, fractal analysis is the most common method for the assessment of bone structure from plain radiographs.^3^,^7^,^11^,^12^,^16^,^17^,^19^,^20^,^26^ To date, bone texture or density has not been assessed from clinical X-ray images with minimal PP and compared between controls and OA subjects. We believe that simultaneous assessment of bone density and structure from a plain radiograph would be an advantage. Furthermore, we also believe that especially in multicenter studies, the results would be more comparable if the effect of PP algorithms is minimized, i.e., by calculating the bone density and texture from X-ray images with minimal possible PP strength, or using an identical PP algorithms for the images.

Consequently, the first aim of this study was to investigate the relationship of radiography-based bone density and texture between X-ray images with minimal PP and with default clinical PP algorithm to find out how much the PP algorithm affect these measurements. The second aim was to compare the differences in bone characteristics (density and texture) between controls and subjects with knee OA or medial tibial BMLs to find out whether the changes in bone characteristics can be detected from X-ray image with minimal PP. Finally, a machine learning model was built to assess how well subjects with and without OA or medial tibial BMLs can be discriminated based on their bone density and texture (from X-ray image with minimal PP) only.

## Subjects and Methods

### Study Subjects

This cross-sectional study included 109 subjects (66 women, 43 men) with and without OA (Table 1). Written informed consent was obtained from each participant. The study was carried out in accordance with the Declaration of Helsinki and approved by the Ethical Committee of Northern Ostrobothnia Hospital District, Oulu University Hospital (number 7/2016).

**Table 1:** Description of the subjects (*n* = 109).

Parameter	Mean (SD)	Min–max
Anthropometric variables		
Age (years)	58.1 (6.0)	45–68
Height (m)	1.70 (0.09)	1.50–1.92
Weight (kg)	78.3 (14.2)	50.0–127.6
Body mass index (kg/m^2^)	27.2 (4.4)	19.7–40.3
KL grade distribution		
KL 0	14	
KL 1	43	
KL 2	28	
KL 3	22	
KL 4	2	

### Acquisition and Grading of the Radiographs

Bilateral posterior-anterior weight-bearing radiographs with knees in semi-flexion were acquired (DigitalDiagnost, Philips Medical Systems, 10° X-ray beam angle, 60 kVp, automatic exposure, pixel size: 0.148 mm × 0.148 mm, source—detector distance: 153 cm) and processed with minimal PP and default clinical PP algorithm. Right knees of the subjects were used in the analyses. Three radiographs with minimal PP and two radiographs with default clinical PP were missing and, thus, the total numbers of radiographs with minimal and clinical PP were 106 and 107, respectively.

An experienced musculoskeletal radiologist (initials: JN) classified the knees according to the KL grading,^13^ in which grade zero corresponds to a healthy knee and grade four to severe OA.

### Selection of Regions of Interests

**Figure 1:**
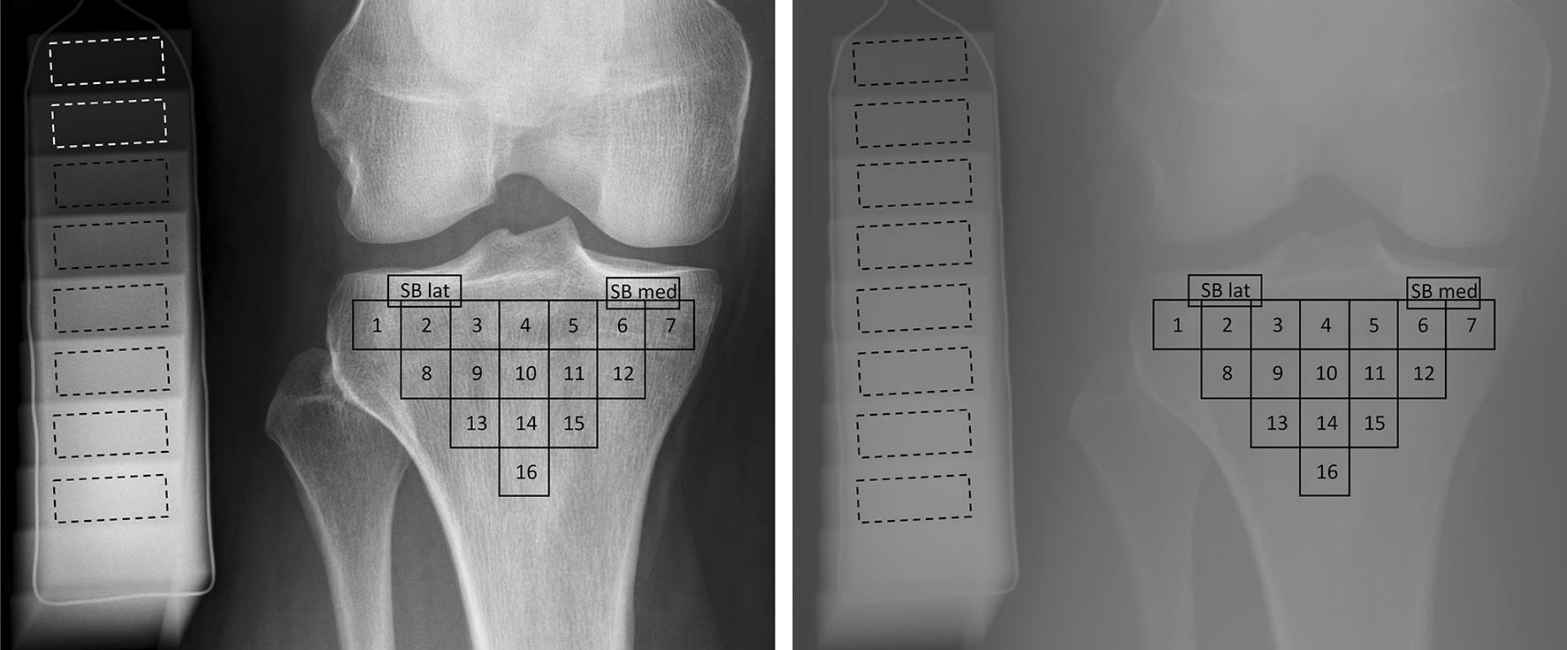
Location of regions of interest (ROIs). The ROIs were exactly in the same location in images with default clinical post-processing (left) and with minimal post-processing (right). Two ROIs were placed in subchondral trabecular bone immediately below the cartilage-bone interface in the middle part of the medial and lateral tibial plateaus. Sixteen ROIs were placed under the dense subchondral trabecular bone area. Dashed rectangles show the areas where the mean value of the steps of the aluminum step wedge were calculated.

To assess bone density and texture from the radiographs, 18 ROIs were semi-automatically placed across the proximal tibia (Fig. 1). The locations were identical in radiographs with minimal and default PP. Two ROIs (size: 14 mm × 6 mm) were placed into the subchondral bone in the middle of the medial and lateral tibial plateaus immediately below the cartilage—bone interface. Anatomical landmarks for the ROIs were tibial spine, subchondral bone plate, the dense subchondral trabecular bone, and outer borders of the proximal tibia. The locations and sizes of the ROIs were based on the previous literature.^7^,^8^,^10^–^12^ A custom-made MATLAB software (version R2017b, The MathWorks, Inc., Natick, MA, USA) was used for the placement (initials: JH) of the ROIs. We have previously shown that the reproducibility of the texture variables from the tibial subchondral and trabecular bone is high.^8^,^10^

### Bone Density Assessment

Two different methods to evaluate bone density were used, i.e., (1) the mean grayscale value of the ROI (= GV) and (2) the aluminum step wedge thickness that corresponds to the measured GV (= GV_mmAl_). The corresponding step wedge thickness was calculated by fitting a third order polynomial to the mean grayscale values of the eight first steps in the step wedge in each image and comparing the values of that fitted curve to the GV. The two thickest steps were omitted because the grayscale values were saturated at those steps. The step wedge was present in all images. For one subject, mean GV in medial subchondral bone ROI was higher than the highest grayscale value in step wedge and that ROI was therefore excluded from the analyses (extrapolation of the step wedge values would have been needed).

### Bone Texture

Fractal signature analysis (FSA) method was used to estimate fractal dimension.^19^,^20^ In brief, the image was dilated and eroded in horizontal and vertical directions with a rod-shaped one-pixel wide structuring element. After that, the volume, *V*, between dilated and eroded images was calculated. Calculations were repeated by varying the element length *r* from 2 to 7 pixels. The surface area, *A*(*r*), was obtained from the Eq. (1):

1 $$A(r) = (V(r) - V(r - 1))/2,$$

Subsequently, a log–log plot was constructed by plotting log of *A*(*r*) against log of *r*. Finally, the fractal dimension was estimated by fitting a regression line to points in the plot and local fractal dimensions were obtained at 0.30, 0.44, 0.59, and 0.74 mm sizes. When the structuring element is pointing in the horizontal direction, fractal dimension of vertical structures (FD_Ver_) is produced and *vice versa*. High fractal dimension values are associated with high complexity of the image, whereas low complexity results in low fractal dimension values.

### Magnetic Resonance Imaging

Right knees of all but one subjects (*n* = 108) were scanned with a 3-Tesla magnetic resonance imaging (MRI) scanner (Siemens Skyra, Siemens Healthcare) using sagittal T2-weighted dual-echo steady-state (repetition time (TR): 14.1 ms, echo time (TE): 5 ms, echo train length (ETL): 2, pixel size: 0.6 mm × 0.6 mm, slice thickness: 0.6 mm), 3-D sagittal proton-density (PD)-weighted SPACE fat-suppressed turbo spin-echo (TSE) (TR: 1200 ms, TE: 26 ms, ETL: 49, pixel size: 0.6 mm × 0.6 mm, slice thickness: 0.6 mm), coronal PD-weighted TSE (TR: 2800 ms, TE: 33 ms, ETL: 4, pixel size: 0.4 mm × 0.4 mm, slice thickness: 3 mm), and coronal T1-weighted TSE (TR: 650 ms, TE: 18 ms, ETL: 2, pixel size: 0.4 mm × 0.4 mm, slice thickness: 3 mm) sequences. An experienced musculoskeletal radiologist (initials: JN) assessed the presence of BMLs and a subject was included in the medial tibial BML group if he/she had any BML (including ill-defined lesions, bone marrow edema and subchondral cysts) in the medial anterior, central, or posterior part of tibia.

### Statistical Analyses

The normality of the variables was assessed using Shapiro–Wilk test. The relationship between normally distributed variables was evaluated using Pearson’s correlation analysis (*r*) while Spearman’s rank correlation (*r*_s_) was applied if at least one of the variables was not normally distributed. Absolute values of correlation coefficients were interpreted as follows: 0.00–0.19 very weak, 0.20–0.39 weak, 0.40–0.59 moderate, 0.60–0.79 strong and 0.80–1.00 very strong correlation.^30^ No adjustments for multiple comparisons were performed.^28^

For comparing differences between controls (group 0, KL < 2), subjects with radiographic knee OA (KL ≥ 2) without medial tibial BML (group 1), and subjects with medial tibial BML (group 2), based on the normality of the variables either analysis of variance (ANOVA) or Kruskal–Wallis test was applied. These analyses were combined with *post hoc* tests without correction for the Type I error rate across the pairwise tests and using Bonferroni correction. Clinical covariates were age, gender, and body mass index. Bone characteristics from X-rays images with minimal PP was used.

Machine learning was used for dimensionality reduction and to assess how well subjects with (KL ≥ 2) and without OA (KL < 2) or BMLs can be discriminated based on their bone density and texture (from X-ray images with minimal PP) only. For this, a regularized logistic regression method called elastic net was used.^6^,^35^ The elastic net linearly combines the L1 and L2 penalties of lasso and ridge regression methods. To optimize the ratio of the L1 and L2 penalties (*α*) and the strength of the penalty parameter (*λ*) of the elastic net, leave-one-out cross-validation (analyses were repeated so many times that each sample was once in the validation set while the rest of the samples were used for training) with a grid search was performed. In the grid, the values of α varied from 0.1 to 1 with an increment of 0.05 and *λ* from 0.001 to 0.15 with an increment of 0.009. When *α* is close to zero, the elastic net approaches ridge regression, while when *α* is 1, lasso regression is performed. In cross-validation, the performance of the bone density and texture (from X-ray images with minimal PP) feature model to discriminate subjects with and without OA as well as subjects with and without medial tibial BMLs was assessed using area under the receiver operating characteristics curve (ROC AUC). Statistical analyses and elastic net experiments were done using R (version 3.1.2) software with Caret^18^ (version 6.0), pROC^27^ (version 1.8), glmnet^6^ (version 2.0), and dunn.test (version 1.3.2) packages.

## Results

### Comparison of Bone Density and Texture Between Minimal and Default Clinical PP

**Figure 2:**
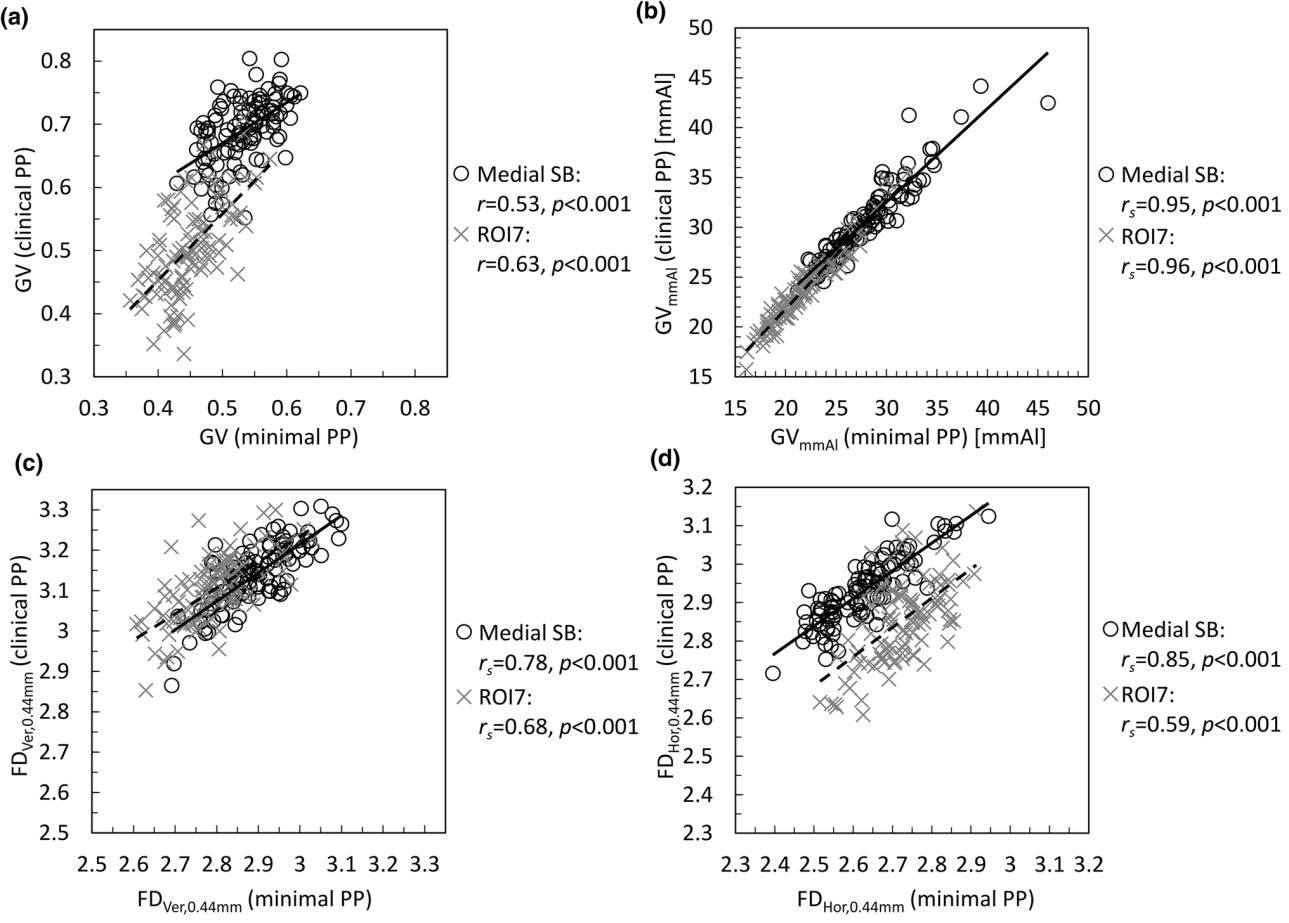
Correlations between (a) GV, (b) GV_mmAl_, (c) FD_Ver,0.44mm_, and (d) FD_Hor,0.44mm_ measured from X-ray images with minimal and default clinical post-processing (PP) in medial subchondral bone (SB) and ROI7. The scale varies between figures but is constant within a figure.

Without normalization of grayscale values in the reference step wedge, the correlations between GVs from X-ray images with minimal PP and default clinical PP varied from 0.18 (*p *= 0.07) to 0.63 (*p *< 0.001) depending on the ROI (Fig. 2, Supplementary Table 5). For the GV_mmAl_ variable, statistically significant (*p *< 0.001) very strong correlations were found in all ROIs (between 0.94 and 0.97) (Fig. 2, Supplementary Table 5).

For the FD_Ver_ texture variable, strong to very strong correlations (between 0.62 and 0.97, *p *< 0.001) were found between X-ray images with minimal PP and default clinical PP at all scales and ROIs (Fig. 2, Supplementary Table 5), except FD_Ver,0.59mm_ in ROI7 (0.51, *p *< 0.001). For FD_Hor_, the correlations varied at different scales and ROIs from no correlation to very strong correlation (between − 0.10 and 0.97) (Fig. 2, Supplementary Table 5). The correlation coefficients were especially low for FD_Hor,0.74mm_ (between − 0.10 and 0.49).

### Differences in Bone Characteristics Between Controls, OA Subjects, and Subjects with Medial Tibial BMLs

Subjects with medial tibial BMLs (group 2) had significantly (*p *< 0.05) higher body mass index than subjects with OA but without BMLs (group 1) or controls (Table 2). Moreover, subjects with medial tibial BMLs were older (*p *< 0.05) than controls.

**Table 2:** Mean (standard deviation) values of the selected variables among controls, subjects with radiographic OA but no medial tibial BMLs, and subjects with medial tibial BMLs.

Variable	Group 0: Controls (*n* = 52)	Group 1: OA, no medial tibial BML (*n* = 30)	Group 2: Medial tibial BML (*n* = 23)	*p* value
Age (years)	56.4 (6.3)^**2**^	58.3 (5.5)	60.8 (4.4)	0.019^a^
Body mass index (kg/m^2^)	25.0 (2.5)^**1,2**^	28.1 (3.8)^**2**^	30.9 (5.8)	< 0.001
GV_mmAl_ in medial SB (mmAl)	26.9 (3.1)^**1,2**^	29.2 (4.7)	29.6 (4.5)	0.011^a^
GV_mmAl_ in ROI6 (mmAl)	25.0 (2.6)^**1**,2^	26.8 (3.4)	26.8 (3.9)	0.016
GV_mmAl_ in ROI7 (mmAl)	20.1 (2.1)^**1**,**2**^	22.3 (2.9)^2^	24.0 (4.0)	< 0.001
GV_mmAl_ in ROI12 (mmAl)	23.6 (2.4)^**1**,**2**^	25.4 (2.9)	25.6 (3.7)	0.006
FD_Ver,0.30mm_ in medial SB	2.65 (0.09)^**2**^	2.68 (0.08)	2.71 (0.07)	< 0.001
FD_Ver,0.30mm_ in ROI6	2.65 (0.08)^**2**^	2.66 (0.07)	2.70 (0.06)	0.028
FD_Ver,0.30mm_ in ROI7	2.55 (0.06)^**2**^	2.57 (0.06)^**2**^	2.62 (0.06)	< 0.001^a^
FD_Ver,0.30mm_ in ROI12	2.70 (0.06)^**2**^	2.71 (0.06)	2.74 (0.06)	0.043
FD_Ver,0.30mm_ in ROI15	2.70 (0.06)^**2**^	2.72 (0.08)^**2**^	2.74 (0.05)	0.013^a^
FD_Ver,0.44mm_ in medial SB	2.86 (0.09)^1,**2**^	2.90 (0.08)^2^	2.95 (0.08)	< 0.001
FD_Ver,0.44mm_ in ROI7	2.76 (0.08)^**1**,**2**^	2.80 (0.09)^2^	2.85 (0.08)	< 0.001^a^
FD_Ver,0.59mm_ in medial SB	2.90 (0.11)^1,**2**^	2.96 (0.12)	3.01 (0.11)	< 0.001
FD_Ver,0.59mm_ in ROI7	2.79 (0.11)^1,**2**^	2.85 (0.12)^2^	2.92 (0.12)	< 0.001
FD_Ver,0.59mm_ in ROI12	3.14 (0.10)^1,**2**^	3.19 (0.12)	3.20 (0.07)	0.024
FD_Ver,0.74mm_ in medial SB	2.84 (0.11)^**2**^	2.90 (0.16)^2^	2.96 (0.13)	0.002^a^
FD_Ver,0.74mm_ in ROI7	2.70 (0.15)^1,**2**^	2.77 (0.15)	2.85 (0.17)	< 0.001
FD_Ver,0.74mm_ in ROI12	3.08 (0.13)^**1**,**2**^	3.16 (0.13)	3.17 (0.13)	0.005
FD_Hor,0.30mm_ in lateral SB	2.52 (0.08)^2^	2.55 (0.10)	2.57 (0.08)	0.046
FD_Hor,0.30mm_ in ROI7	2.57 (0.08)^**2**^	2.59 (0.08)	2.62 (0.07)	0.025
FD_Hor,0.59mm_ in ROI2	2.96 (0.07)	2.98 (0.05)^**2**^	2.93 (0.10)	0.020
FD_Hor,0.59mm_ in ROI3	2.98 (0.07)^**2**^	2.97 (0.07)^**2**^	2.91 (0.09)	< 0.001^a^
FD_Hor,0.74mm_ in ROI1	2.92 (0.06)^**2**^	2.92 (0.08)^**2**^	2.87 (0.08)	0.013
FD_Hor,0.74mm_ in ROI2	2.97 (0.08)^2^	2.98 (0.14)^2^	2.91 (0.14)	0.040
FD_Hor,0.74mm_ in ROI3	2.98 (0.07)^**2**^	2.97 (0.11)^**2**^	2.91 (0.11)	0.004^a^
FD_Hor,0.74mm_ in ROI7	2.77 (0.08)^**2**^	2.75 (0.11)^2^	2.70 (0.11)	0.012^a^

Bone density and texture variables were measured from X-ray images with minimal post-processing

*SB* subchondral bone, *ROI* region of interest, *GV*_*mmAl*_ mean grayscale value calibrated with aluminum step wedge, *FD* fractal dimension of vertical (Ver) or horizontal (Hor) structures, ^a^differences tested using Kruskal–Wallis test. Numbers in superscript means significant differences between groups without correction of *p*-values. Bolded numbers means significant differences between groups using Bonferroni *post hoc* test

GV_mmAl_ from X-ray images with minimal PP was significantly (*p *< 0.05) higher in group 1 (OA without medial tibial BML) and in group 2 (medial tibial BML) than in control group in all medial side ROIs (subchondral bone ROI and ROI6, ROI7, and ROI12) (Table 2).

Statistically significant differences (*p *< 0.05) in FD_Ver_ (in all scales) from X-ray images with minimal PP in medial side ROIs were found. For example, FD_Ver,0.44mm_ in subchondral bone and in ROI7 was significantly different among controls than in group 1 (OA without medial tibial BML) or group 2 (medial tibial BML) (Table 2). Statistically significant differences (*p *< 0.05) in FD_Hor_ were found in medial and lateral side ROIs (Table 2).

### Classification of OA or BML Subjects and Controls

**Figure 3:**
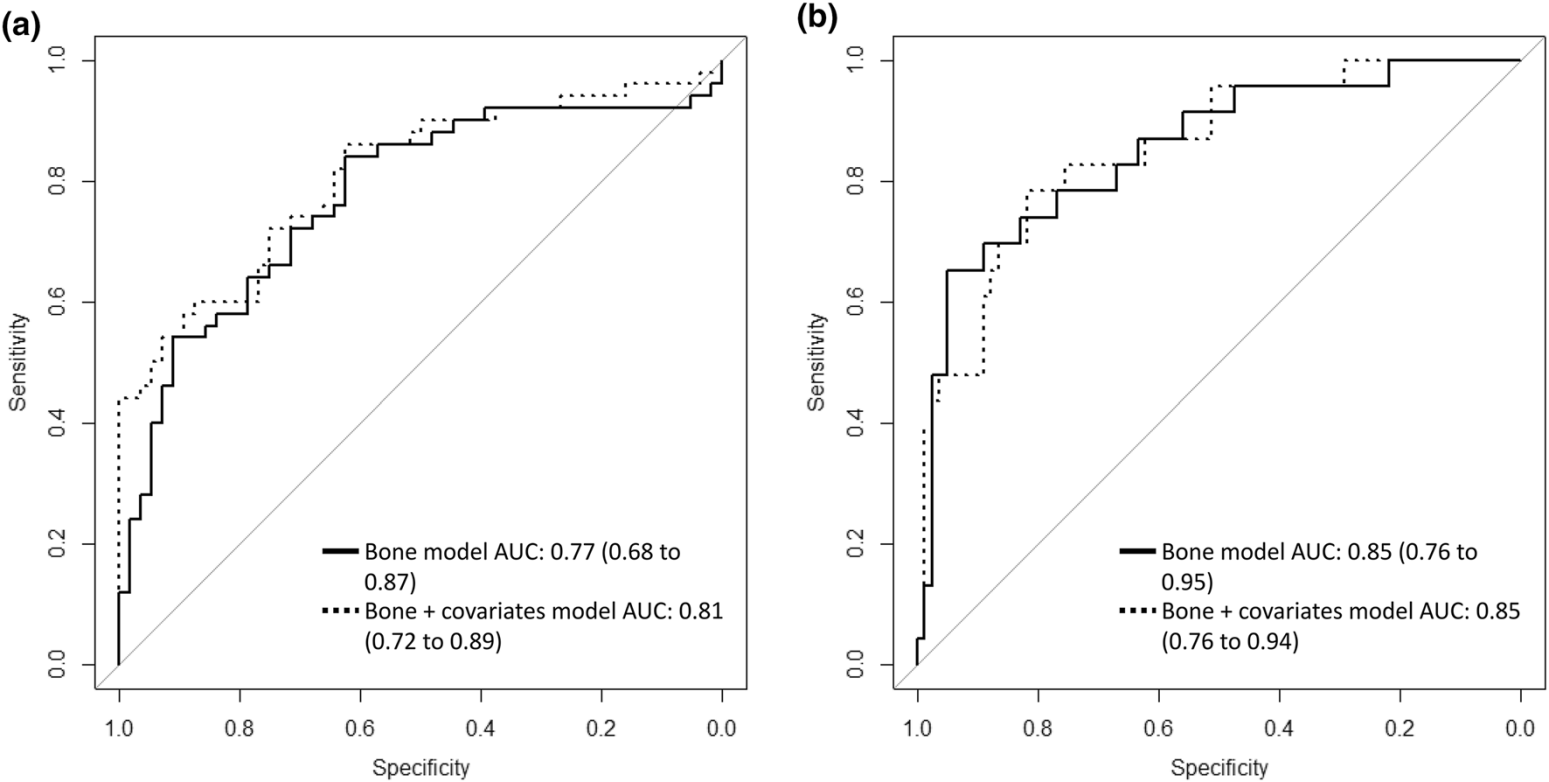
Receiver operating characteristics curves and respective area under the curve (AUC) values for discriminating (a) subjects without and with radiographic knee osteoarthritis as well as (b) subjects without and with medial tibial bone marrow lesions using models that included bone characteristics (bone density and texture) from X-ray images with minimal post-processing and bone characteristics combined with covariates (age, gender, body mass index).

A ROC AUC value of 0.77 (95% confidence interval (CI) 0.68–0.87) was obtained for classifying healthy and OA subjects using the elastic model with variables describing bone density and texture from X-ray image with minimal PP (Fig. 3a). The values for *α* and *λ* hyperparameters of the elastic model were 1 and 0.118, respectively. The bone density and texture variables that were selected in the final model are shown in Table 3. A ROC AUC value of 0.81 (95% CI 0.72–0.89) was obtained when covariates (age, gender, and body mass index) were included in the model (Fig. 3a).

**Table 3:** Bone density and texture variables from X-ray images with minimal post-processing in the elastic net model to discriminate healthy (*n* = 56) and subjects with radiographic knee osteoarthritis (*n* = 50).

Variable	Coefficient
Intercept	− 0.111
GV_mmAl_ in ROI7	0.470
FD_Ver,0.59mm_ in medial SB	0.003
FD_Ver,0.44mm_ in ROI7	0.174

*SB* subchondral bone, *ROI* region of interest, *GV*_*mmAl*_ mean grayscale value calibrated with aluminum step wedge, *FD*_*Ver*_ fractal dimension of vertical structures

A ROC AUC value of 0.85 (95% CI 0.76–0.95) was obtained for classifying controls and subjects with medial tibial BML using the elastic model with variables describing bone density and texture (Fig. 3b). The values for *α* and *λ* hyperparameters of the elastic model were 0.8 and 0.037, respectively. The bone density and texture variables that were selected in the final model are shown in Table 4. A similar ROC AUC value of 0.85 (95% CI 0.76–0.94) was obtained when covariates were included in the model (Fig. 3b).

**Table 4:** Bone density and texture variables from X-ray images with minimal post-processing in the elastic net model to discriminate subjects without (*n* = 82) and with medial tibial bone marrow lesion (*n* = 23).

Variable	Coefficient
Intercept	− 1.752
GV_mmAl_ in ROI7	0.220
FD_Ver,0.44mm_ in medial SB	0.396
FD_Ver,0.74mm_ in medial SB	0.004
FD_Ver,0.30mm_ in ROI7	0.260
FD_Ver,0.30mm_ in ROI12	0.127
FD_Ver,0.30mm_ in ROI15	0.393
FD_Ver,0.59mm_ in ROI7	0.197
FD_Ver,0.74mm_ in ROI4	− 0.092
FD_Ver,0.74mm_ in ROI6	− 0.126
FD_Hor,0.59mm_ in ROI2	− 0.012
FD_Hor,0.59mm_ in ROI3	− 0.644
FD_Hor,0.59mm_ in ROI13	− 0.091
FD_Hor,0.74mm_ in ROI1	− 0.351
FD_Hor,0.74mm_ in ROI2	− 0.012
FD_Hor,0.74mm_ in ROI5	− 0.313
FD_Hor,0.74mm_ in ROI7	− 0.213
FD_Hor,0.74mm_ in ROI8	− 0.443
FD_Hor,0.74mm_ in ROI12	− 0.243

*SB* subchondral bone, *ROI* region of interest, *GV*_*mmAl*_ mean grayscale value calibrated with aluminum step wedge, *FD* fractal dimension of vertical (Ver) or horizontal (Hor) structures

## Discussion

This study evaluated bone density and texture from knee X-ray images with minimal PP. First, the association of bone density and texture between X-ray images with minimal PP and default clinical PP was assessed. Our results show that bone density was strongly correlated between these two PP methods when the grayscale values were calibrated with the reference step wedge. Correlations of bone texture parameters, on the other hand, varied from weak to very strong. Second, we assessed bone density and bone texture from X-ray images with minimal PP, and significant differences between controls (group 0), subjects with OA but without medial tibial BMLs (group 1), and subjects with medial tibial BMLs (group 2) were found. Third, machine learning based elastic net model showed that both bone density and texture parameters from X-ray images with minimal PP contributed to the model when discriminating controls and subjects with OA or subjects with BMLs. Furthermore, relatively good ROC AUC values to discriminate subjects without and with OA (0.77), as well as without and with BMLs (0.85), using bone density and texture parameters were obtained.

Strong associations were obtained when the grayscale values were calibrated, whereas the correlations between the grayscale values without calibration were weak or moderate. Based on this and earlier results, calibration of grayscale values are required when assessing bone density from plain radiographs.^9^,^14^ Varying correlations in texture variables between X-ray images were found. In general, for example resolution and structures in an image affect fractal dimension values. One reason for varying correlations may be that the clinical PP algorithm applies non-linear filtering and adjusts contrast curves of an image and, for example, edges in the image are enhanced. The appearance of the bone contours and trabeculae was visually different between these two images. The lower correlation were found especially in FD_Ver_ and FD_Hor_ parameters at larger scales (0.59 or 0.74 mm) and may be due to different appearance of the bone trabeculae. Our results indicate that when assessing bone texture at larger scales, the effect of PP should be considered especially if the images come from X-ray imaging systems from different manufacturers.

Differences in bone density and texture between controls and subjects with OA without medial tibial BMLs as well as subjects with medial tibial BMLs were found. Bone density was higher among subjects with OA and among subjects with BMLs than among controls in medial side ROIs. Bone sclerosis is most probably the reason for the higher bone density values. Differences in bone texture between groups using FD_Ver_ was observed in medial side ROIs while FD_Hor_ was significantly different in some lateral side ROIs, too. These results show that the bone structure was different between groups. In our earlier study, we showed that FD_Ver_ was associated with 3-dimensional connection and separation of the bone trabeculae.^9^ The finding that bone density and texture differs between controls and OA subjects is in line with previous studies using plain knee radiographs with clinical PP algorithm.^3^,^8^,^19^,^22^,^23^,^25^ The finding for the bone density, however, contradicts for one study in which no association between KL grade and radiography-based bone density in knee was found.^15^ Our previous study revealed that bone texture assessed from radiographs differs between subjects with and without bone marrow lesions (BMLs), but bone density was not assessed in that study.^7^ In general, our present results demonstrate that bone density and texture can be assessed from X-ray images with minimal PP to detect differences between controls, subjects with OA, and subjects with BMLs.

To our knowledge, this is the first study that assessed bone density and texture from X-ray images with minimal PP among subjects with OA or BMLs. Because the direct evaluation of grayscale values of a radiograph is problematic, calibration of the grayscale values using an aluminum step wedge has been proposed.^9^,^14^,^22^,^34^ In an earlier study, bone density in human cadaver tibia was assessed from X-ray image with minimal PP and a strong correlation to actual bone mineral density assessed with dual X-ray absorptiometry was reported.^14^ Another study with human cadaver tibias showed that radiography-based tibial bone density and texture are related with the actual 3-dimensional structure and amount of bone.^9^

Elastic net models were used to assess how well subjects with and without OA or BMLs can be discriminated based on their bone density and texture. Leave-one-out cross-validation was used in order to find optimal hyperparameters for the models. The elastic net also reduces the dimensionality of the feature vector, which was necessary because initially all bone density and texture variables from all ROIs were fed into the model. The ROC AUC values to discriminate subjects without and with OA (0.77) as well as without and with medial tibial BMLs (0.85) using bone density and texture variables were relatively high. When covariates were included in the model, the classification performance was slightly improved in discriminating subjects without and with OA (ROC AUC: 0.81, body mass index and age in the model), but not for discriminating subjects without and with BMLs (ROC AUC: 0.85, body mass index in the model). The results are in line with previous studies, although they used plain knee radiographs with clinical PP algorithm.^31^,^33^ One study reported an accuracy of 85.4% (87.0% sensitivity, 83.8% specificity) for discriminating healthy and OA subjects using bone texture from plain knee radiographs.^33^ They used signature dissimilarity method to obtain bone texture. Another study reported a ROC AUC of 0.74 for discriminating healthy and OA subjects using directional fractal signature method.^31^

It should be noted that a perfect classification was not expected in this study. This is because bone texture does not actually directly affect the KL grading, yet marginal osteophytes, bone sclerosis, cysts, deformation of bone, and narrowing of the joint space are considered in it. It should also be mentioned that sensitivities and specificities of 54–66 and 64–78%, respectively, for OA classification have been reported, when comparing clinical OA (by assessing subject’s medical history, symptoms, and physical examination) and radiographic OA (controls: KL < 2, OA: KL ≥ 2) assessments and using clinical OA as a reference.^5^, ^24^ Furthermore, BMLs were assessed from MRI data. Thus, it can be that some subjects with OA do not actually have changes in their subchondral or trabecular bone. The use of KL grade as ground truth was justified because it is the gold standard when assessing the level of radiographic OA. When aiming to automatically assess the KL grade, the entire joint area should be fed in the model.^1^,^2^,^29^,^32^ However, in this study we wanted to specifically evaluate the changes in bone density and texture.

This study has some limitations. First, bone density and texture variables are quantitative and continuous, whereas KL grading and BML evaluation are semi-quantitative, subjective, and discrete. Furthermore, bone texture is not directly evaluated in KL grading. Second, due to restrictions of our sample size, KL0 and KL1 grades were considered as controls, although KL1 subjects can also be considered as doubtful OA and they might have some OA-related changes. Furthermore, because of the limited sample size, we did not assess the performance of elastic net models to classify OA subjects with and without BMLs. Third, our data was cross-sectional and, thus, we were unable to assess how well bone density and texture predict the development or progression of OA. Fourth, in future, studies with higher number of X-rays from different X-ray imaging systems with minimal PP are desired.

In conclusion, PP algorithm did have effect on the grayscale values and texture variables, especially on fractal dimensions with larger scales. Differences in bone density and texture, assessed from X-ray images with minimal PP, were found between controls, subjects with OA but without BMLs, and subjects with medial tibial BMLs. Finally, relatively good classification between controls and OA subjects as well as controls and subjects with medial tibial BML using only bone density and texture variables was obtained. Our results indicate that calibration of grayscale values are required when assessing bone density from plain radiographs, and the effect of PP should be considered when assessing bone texture at larger scales.

## Electronic Supplementary Material

Below is the link to the electronic supplementary material.

Supplementary material 1 (DOCX 73 kb)
