# Supplementary material for: Bone Density and Texture from Minimally Post-Processed Knee Radiographs in Subjects with Knee Osteoarthritis
Source: Ann Biomed Eng. 2019 Feb 14;47(5):1181–90. doi: 10.1007/s10439-019-02227-y (PMC6453872; doi:10.1007/s10439-019-02227-y)
Supplement: Supplementary file 1 — Supplementary material 1 (DOCX 73 kb) [file 10439_2019_2227_MOESM1_ESM.docx]

**Supplementary Table 5.** Correlations between bone characteristics (density and texture variables) measured from X-ray images with minimal and default clinical post-processing. *n* = 103 – 104.

| ROI | GV | GV_mmAl_ | FD_Ver,0.30mm_ | FD_Ver,0.44mm_ | FD_Ver,0.59mm_ | FD_Ver,0.74mm_ | FD_Hor,0.30mm_ | FD_Hor,0.44mm_ | FD_Hor,0.59mm_ | FD_Hor,0.74mm_ |
| --- | --- | --- | --- | --- | --- | --- | --- | --- | --- | --- |
| SB medial | 0.53** | 0.95** | 0.81** | 0.78** | 0.78** | 0.66** | 0.91** | 0.85** | 0.88** | -0.10 |
| SB lateral | 0.39** | 0.94** | 0.94** | 0.87** | 0.81** | 0.85** | 0.95** | 0.89** | 0.90** | 0.25** |
| ROI1 | 0.23* | 0.94** | 0.94** | 0.87** | 0.77** | 0.70** | 0.96** | 0.62** | 0.35** | 0.24* |
| ROI2 | 0.36** | 0.96** | 0.96** | 0.97** | 0.93** | 0.90** | 0.97** | 0.68** | 0.52** | 0.48** |
| ROI3 | 0.46** | 0.96** | 0.96** | 0.94** | 0.91** | 0.90** | 0.82** | 0.56** | 0.25* | 0.13 |
| ROI4 | 0.47** | 0.97** | 0.96** | 0.97** | 0.96** | 0.94** | 0.72** | 0.64** | 0.43** | 0.35** |
| ROI5 | 0.42** | 0.97** | 0.94** | 0.96** | 0.95** | 0.93** | 0.73** | 0.66** | 0.47** | 0.45** |
| ROI6 | 0.41** | 0.96** | 0.97** | 0.95** | 0.93** | 0.90** | 0.76** | 0.66** | 0.49** | 0.35** |
| ROI7 | 0.63** | 0.96** | 0.88** | 0.68** | 0.51** | 0.64** | 0.96** | 0.59** | 0.29** | 0.13 |
| ROI8 | 0.32** | 0.96** | 0.96** | 0.97** | 0.95** | 0.94** | 0.76** | 0.61** | 0.33** | 0.23* |
| ROI9 | 0.36** | 0.97** | 0.96** | 0.95** | 0.95** | 0.95** | 0.70** | 0.70** | 0.48** | 0.40** |
| ROI10 | 0.35** | 0.97** | 0.94** | 0.97** | 0.97** | 0.94** | 0.67** | 0.69** | 0.51** | 0.42** |
| ROI11 | 0.29** | 0.97** | 0.96** | 0.93** | 0.89** | 0.86** | 0.65** | 0.72** | 0.50** | 0.42** |
| ROI12 | 0.33** | 0.96** | 0.93** | 0.86** | 0.70** | 0.62** | 0.60** | 0.74** | 0.46** | 0.30** |
| ROI13 | 0.39** | 0.97** | 0.95** | 0.94** | 0.96** | 0.95** | 0.69** | 0.75** | 0.61** | 0.49** |
| ROI14 | 0.33** | 0.96** | 0.95** | 0.97** | 0.95** | 0.95** | 0.69** | 0.66** | 0.55** | 0.47** |
| ROI15 | 0.18 | 0.97** | 0.92** | 0.94** | 0.90** | 0.82** | 0.77** | 0.71** | 0.49** | 0.48** |
| ROI16 | 0.27** | 0.95** | 0.95** | 0.96** | 0.96** | 0.95** | 0.60** | 0.68** | 0.46** | 0.29** |
| ***p* < 0.001, **p* < 0.05, ROI = region of interest, SB = subchondral bone, GV = mean grayscale value of the ROI, GV_mmAl_ = GV calibrated with aluminum step wedge, FD = fractal dimension of vertical (Ver) or horizontal (Hor) structures. | | | | | | | | | | |

**Supplementary Table 6**. Variables in the final elastic net model to discriminate healthy (*n* = 56) and subjects with radiographic knee osteoarthritis (*n* = 50) using covariates and bone characteristics (density and texture) from X-ray images with minimal post-processing. The values for α and λ hyperparameters of the elastic model were 0.35 and 0.136, respectively.

| Variable | Coefficient |
| --- | --- |
| Intercept | -0.064 |
| GV_mmAl_ in SB | 0.068 |
| GV_mmAl_ in ROI7 | 0.252 |
| FD_Ver,0.59mm_ in medial SB | 0.053 |
| FD_Ver,0.74mm_ in medial SB | 0.073 |
| FD_Ver,0.30mm_ in ROI7 | 0.052 |
| FD_Ver,0.44mm_ in ROI7 | 0.133 |
| FD_Ver,0.44mm_ in ROI13 | 0.043 |
| FD_Ver,0.44mm_ in ROI14 | 0.061 |
| FD_Ver,0.59mm_ in ROI3 | -0.018 |
| FD_Ver,0.59mm_ in ROI7 | 0.021 |
| FD_Ver,0.59mm_ in ROI13 | 0.104 |
| FD_Ver,0.59mm_ in ROI14 | 0.013 |
| FD_Ver,0.74mm_ in ROI3 | -0.089 |
| FD_Ver,0.74mm_ in ROI7 | 0.075 |
| FD_Ver,0.74mm_ in ROI8 | -0.000 |
| FD_Ver,0.74mm_ in ROI12 | -0.117 |
| FD_Hor,0.30mm_ in ROI16 | -0.047 |
| FD_Hor,0.44mm_ in ROI6 | 0.092 |
| FD_Hor,0.59mm_ in ROI3 | -0.062 |
| FD_Hor,0.74mm_ in ROI7 | -0.197 |
| FD_Hor,0.74mm_ in ROI15 | 0.000 |
| Age | 0.137 |
| Body mass index | 0.453 |
| SB = subchondral bone, ROI = region of interest, GV_mmAl_ = mean grayscale value calibrated with aluminum step wedge, FD = fractal dimension of vertical (Ver) or horizontal (Hor) structures. | |

**Supplementary Table 7.** Variables in the elastic net model to discriminate subjects without (*n* = 82) and with medial tibial bone marrow lesion (*n* = 23) using covariates and bone characteristics (density and texture) from X-ray images with minimal post-processing. The values for α and λ hyperparameters of the elastic model were 0.8 and 0.019, respectively.

| Variable | Coefficient |
| --- | --- |
| Intercept | -2.262 |
| GV_mmAl_ in ROI16 | -0.088 |
| FD_Ver,0.44mm_ in medial SB | 0.321 |
| FD_Ver,0.59mm_ in lateral SB | -0.227 |
| FD_Ver,0.74mm_ in medial SB | 0.246 |
| FD_Ver,0.74mm_ in lateral SB | -0.050 |
| FD_Ver,0.30mm_ in ROI7 | 0.235 |
| FD_Ver,0.30mm_ in ROI12 | 0.303 |
| FD_Ver,0.30mm_ in ROI15 | 0.650 |
| FD_Ver,0.59mm_ in ROI4 | -0.298 |
| FD_Ver,0.59mm_ in ROI6 | -0.159 |
| FD_Ver,0.59mm_ in ROI7 | 0.241 |
| FD_Ver,0.59mm_ in ROI8 | -0.104 |
| FD_Ver,0.59mm_ in ROI10 | 0.472 |
| FD_Ver,0.59mm_ in ROI12 | 0.027 |
| FD_Ver,0.74mm_ in ROI4 | -0.373 |
| FD_Ver,0.74mm_ in ROI6 | -0.071 |
| FD_Ver,0.74mm_ in ROI14 | -0.030 |
| FD_Hor,0.30mm_ in ROI2 | -0.000 |
| FD_Hor,0.30mm_ in ROI7 | 0.095 |
| FD_Hor,0.59mm_ in ROI3 | -0.674 |
| FD_Hor,0.59mm_ in ROI13 | -0.267 |
| FD_Hor,0.59mm_ in ROI16 | 0.205 |
| FD_Hor,0.74mm_ in ROI1 | -0.518 |
| FD_Hor,0.74mm_ in ROI2 | -0.186 |
| FD_Hor,0.74mm_ in ROI4 | 0.000 |
| FD_Hor,0.74mm_ in ROI5 | -0.349 |
| FD_Hor,0.74mm_ in ROI7 | -0.313 |
| FD_Hor,0.74mm_ in ROI8 | -0.744 |
| FD_Hor,0.74mm_ in ROI12 | 0.478 |
| Body mass index | 0.593 |
| SB = subchondral bone, ROI = region of interest, GV_mmAl_ = mean grayscale value calibrated with aluminum step wedge, FD = fractal dimension of vertical (Ver) or horizontal (Hor) structures. | |
